# Organoprotective Effects of Spironolactone on Top of Ramipril Therapy in a Mouse Model for Alport Syndrome

**DOI:** 10.3390/jcm10132958

**Published:** 2021-06-30

**Authors:** Diana Rubel, Yanqin Zhang, Nenja Sowa, Rainer Girgert, Oliver Gross

**Affiliations:** 1 Clinic of Nephrology and Rheumatology, University Medical Center Goettingen, 37075 Goettingen, Germany; diana@rubel.de (D.R.); yiran6666@126.com (Y.Z.); nenjasowa@googlemail.com (N.S.); rainer.girgert@med.uni-goettingen.de (R.G.); 2 Department of Pediatrics, Peking University First Hospital, Beijing 100034, China

**Keywords:** hereditary kidney disease, progressive kidney fibrosis, nephroprotective therapy, type IV collagen

## Abstract

Angiotensin-converting enzyme inhibitors (ACEi) delay progression of the inherited renal disease Alport syndrome. However, the effect of ACEis weakens gradually due to an “aldosterone escape”. Here, we investigate if an aldosterone antagonist can counteract loss of ACEi-efficacy. *COL4A3−/−* mice were treated with ramipril (ACEi), starting at 4.5 weeks of age, and spironolactone was added at 7 weeks of age. Lifespan until renal failure, as well as kidney function parameters, were investigated. Dual therapy decreased proteinuria levels compared to ACEi monotherapy. Matrix accumulation, as well as tubulointerstitial and glomerular scar-tissue formation, were significantly reduced compared to untreated mice and ACEi-monotherapy at 75 and 100 days. Lifespan in dual treated mice was extended compared to untreated mice. However, lifespan was not superior to ACEi monotherapy–despite improved urea-nitrogen levels in the dual therapy group. In conclusion, adding the aldosterone-antagonist spironolactone to ACEi therapy further improved kidney function and reduced proteinuria and fibrosis. However, survival was not improved further, possibly due to premature death from side effects of dual therapy such as hyperkalemia. Thus, dual therapy could offer an effective therapy option for Alport syndrome patients with progressive proteinuria. However, the risks of adverse events require close monitoring.

## 1. Introduction

Alport syndrome (AS) is an inherited renal disease caused by variants in *COL4A3*, *COL4A4*, or *COL4A5* gene [1,2,3]. The clinical manifestations of Alport syndrome include hematuria, proteinuria, and progressive renal failure, sometimes accompanied by hearing loss and ocular lesions [4]. End-stage renal disease (ESRD) is inevitable in the majority of patients with Alport syndrome and typically occurs during early adulthood. It is reported that 90% of patients develop ESRD by the age of 30 years in males with X-linked Alport syndrome (XLAS) due to variants in *COL4A5* gene. In autosomal recessive Alport syndrome (ARAS) with variants in the *COL4A3* or *COL4A4* genes, female and male patients are equally affected and also reach ESRD early in life [5,6,7,8,9].

In the last two decades, potential therapies preventing or delaying the progression of kidney dysfunction of patients with AS have become a prominent focus of research. Most relevant, blockade of the renin-angiotensin-aldosterone system (RAAS) by angiotensin-converting enzyme inhibitors (ACEi) or angiotensin receptor blockade (ARB) has been shown to delay renal failure and reduce renal fibrosis in COL4A3 knockout mice as animal models of AS [10,11]. Moreover, retrospective analysis of patients with AS strongly suggested that ACEi therapy can delay ESRD by many years [12]. Retrospective data from children with AS showed that proteinuria decreased significantly in the first two years of ACEi and ARB therapy [13,14]. Early RAAS-blockade does not only improve renal survival in patients with AS, but also increases the hope for finding other drugs which work additive to ACEis to further delay disease progression in AS.

In the mouse model for Alport syndrome, some of the positive effects reducing the amount of proteinuria and serum urea of ramipril therapy were lost after several weeks of treatment [10]. Similarly, retrospective data from children with AS indicate that proteinuria gradually increases after three years of ACEi therapy [14]. This negative phenomenon is similar to the ESCAPE trial with aggressive blood pressure control by ramipril in children with chronic kidney disease (CKD), in which some of the early antiproteinuric effects was lost by the third year of follow-up [15]. Experimental data suggest that 30–40% of patients with CKD develop increased levels of plasma aldosterone whilst on long-lasting of ACEi therapy, known as “aldosterone escape”, which correlates with the decline of glomerular filtration rate [16,17].

As a consequence, the “aldosterone escape” is hypothesized to be an important negative pathomechanism for disease progression in AS in the course of long-lasting ACE-inhibition. In the present study, we investigate the hypothesis if the aldosterone antagonist spironolactone can counteract this loss of ACEi-efficacy in the Alport mouse model.

## 2. Materials and Methods

### 2.1. Animals and Treatment

Heterozygous COL4A3 knockout mice (JAX stock #002908, Jackson Laboratories, Bar Harbor, ME, USA) were crossbred on a 129/SvJ genetic background. DNA was isolated from tail biopsies according to manufacturer instruction using Nucleo Spin^®^ Tissue Kit (Macherey Nagel, Düren, Germany). Genotyping of the mice was carried out by PCR with the TopTaq^®^ DNA Polymerase (Qiagen, Hilden, Germany) using a combination of three different Primer (COL4A3 forward 5′-CCAGGCTTAAAGGGAAATCC-3′; COL4A3 reverse 5′-CCTGCTAATATAGGGTTCGAGA-3′; COL4A3 mutant 5′-AAT CGC CAA TGA CAA GAC G-3′). Treatment protocols for the mice were approved by local German authorities and supervised by veterinarians. Sixteen *COL4A3−/−* mice per group were treated with ramipril (Sanofi-Aventis, Paris, France) (ACEi-group) or ramipril plus spironolactone (Sigma Aldrich, S3378, St. Louis, MO, USA). (ACEi+Spiro). Medication was given orally via the drinking water. Ramipril was started in both groups at 4.5 weeks of age with a daily dose of 10 mg/kg/day during the whole experiment. Spironolactone was added to ramipril in the ACEi+Spiro group at 7 weeks of age with a daily dose of 50 mg/kg/day.

Three to four mice per group were sacrificed at two different stages of the disease (75 and 100 days). In the remaining 8 to 9 mice per group, lifespan until renal failure was monitored. Animals were weighed on a weekly basis, and state of health was monitored daily. All results were compared to wildtype mice (WT) and untreated *COL4A3−/−* mice (Plac).

### 2.2. Kidney Function Parameters

Proteinuria was measured in spontaneous urine. Urine samples were processed via chloroform-methanol precipitation and separated by SDS-PAGE using 4–12% Novex Tris Glycine polyacrylamide gradient gels (Life Technologies, Carlsbad, CA, USA). The net intensity of protein bands was measured after Coomassie staining by densitometry using Kodak 1D software. Blood urea nitrogen (BUN) was analyzed using serum samples of sacrificed mice (kinetic UV-Test, Cobas8000 Modular Analyzer Series, Roche Diagnostics, Mannheim, Germany). Serum aldosterone levels were measured by an aldosterone ELISA (catalog no. RE52301, IBL International GmbH, Hamburg, Germany) according to manufacturer instructions.

### 2.3. Immunohistology

Kidneys of sacrificed mice were kept in 4% paraformaldehyde at 40 C overnight and immersion-fixed using HistoCore PEARL (Leica, Wetzlar, Germany). Paraffin-embedded kidneys were sectioned at 3 μm on a Reichert-Jung 2040 Autocut Microtome (Leica Microsystems, Wetzlar, Germany). For staining of laminin 111 and fibronectin, the slides were de-paraffinized and rehydrated. Sections were incubated overnight at 4 °C with the primary antibodies rabbit anti-laminin 111 (1:25, Ab11575, Abcam, Cambridge, UK), and goat anti-fibronectin (1:50, sc6952, Santa Cruz Tech, Dallas, TX, USA). As a negative control, slides were incubated with blocking solution (5% donkey serum in Tris-buffered saline). After washing the samples were stained with the secondary antibody. The Alexa 488-labled antibody goat anti-rabbit (A11008, Lifetech, Waltham, MA, USA) was used to prepare the laminin 111 staining and the donkey anti-goat (A11055, Lifetech, Waltham, MA, USA) was added to the slides for fibronectin staining. Both antibodies incubated for one hour at room temperature in the dark. After washing all slides were mounted in fluorescence mounting medium (S303, Dako, Waldbronn, Germany).

### 2.4. Scoring of Immunohistology

For the glomerular score, images were taken at 400× magnification. For the tubulointerstitial score, a 100× magnification (laminin 111) and 200× magnification (fibronectin) were used. Six images per kidney of three mice per group were scored by three independent individuals in a blinded manner, as previously described [10,11] A pre-defined scoring system from zero (best, normal glomerular or tubular structure) to 3 (worst, severe glomerulosclerosis or tubulointerstitial damage) were used for classification.

### 2.5. Statistical Analysis

Results are presented as mean and standard deviation (SD). One-tailed student T test was used for analyzing differences in body weight. All other data throughout the manuscript were analyzed by two-way analysis of variance (ANOVA) followed by Bonferroni’s multiple comparison test.

## 3. Results

In the present study, we investigate the hypothesis if the aldosterone antagonist spironolactone can counteract this loss of ACEi-efficacy in the Alport mouse model.

### 3.1. Effect on Aldosterone Levels

**Figure 1:**
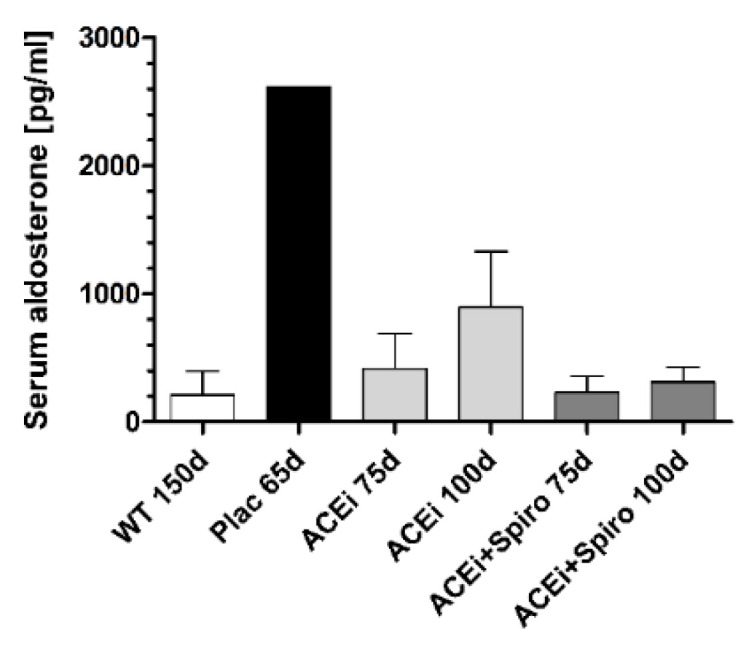
Serum aldosterone levels indicating an aldosterone-escape in Alport mice receiving Angiotensin-converting enzyme inhibitor (ACEi)-monotherapy.

Untreated mice (Plac) showed very high levels of serum aldosterone (2622 pg/mL; Figure 1). In the ACEi group, serum aldosterone decreased to 417 ± 158 pg/mL at 75 days of age and rose to 895 ± 252 pg/mL at 100 days, mimicking an “aldosterone-escape” described in literature [16,17]. When spironolactone was added on top of ramipril, serum aldosterone decreased to 233 ± 62 pg/mL at 75 days and 312 ± 82 pg/mL at 100 days. These levels were in a low range comparable to wild type mice (211 ± 107 pg/mL) and indicated that dual RAAS-blockade (ACEi+Spiro) was effective to test our hypothesis in the following experiments:

### 3.2. Effect on Proteinuria and Kidney Function Parameters

**Figure 2:**
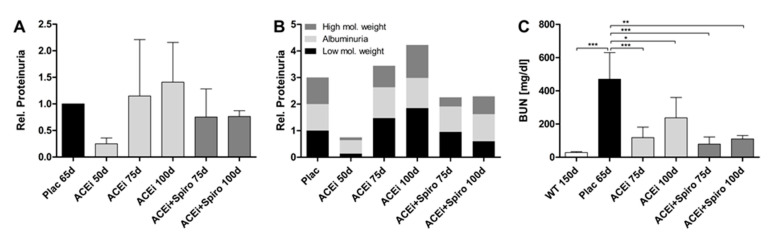
Effect on protein excretion and kidney function on days 50, 75 and 100 of therapy with ACEi and ACEi+Spiro. (**A**) Total renal protein excretion (relative to untreated mice at 65 days) in the ACEi group compared to the ACEi+Spiro group (*n* = 3 for each group); (**B**) Relative change in excretion of high molecular weight proteins (glomerular damage), albumin and low molecular weight proteins (tubular damage) in the ACEi group compared to the ACEi+Spiro group (*n* = 3 for each group); (**C**) Blood urea nitrogen (BUN) levels in the untreated mice compared to the ACEi group and the ACEi+Spiro group (*n* = 3 for WT and ACEi 100d, *n* = 4 for Plac, ACEi 75d and ACEi+Spiro 75d, *n* = 2 for ACEi+Spiro 100d); *** *p* < 0.001; ** *p* < 0.01; * *p* < 0.05.

Kidney function (BUN and proteinuria) was monitored in a total of 17 *COL4A3*−/− mice (4 untreated (Plac), 7 ramipril monotherapy (ACEi), and 6 with dual therapy (ACEi+Spiro)). Differences in proteinuria were not statistically evaluated, because of the semi-quantitative method used and the limited number of mice per group. In ACEi treated mice, urinary protein excretion at 50 days of age was much lower compared to the untreated group (Plac) at 65 days (Figure 2A). However, ACEi monotherapy was not able to maintain its antiproteinuric effect: levels of proteinuria constantly rose despite ACEi therapy at 75 and 100 days of age to levels similar to Alport mice in the placebo group at 65 days. In contrast, dual RAAS-blockade by adding spironolactone on top of ACEi was more effective in preserving the antiproteinuric effect on urinary relative protein excretion (Figure 2A, ACEi+Spiro). Further analysis of the relative amounts of urinary protein showed that albumin excretion levels were comparable in both treated groups compared to placebo group (Figure 2B). In mice treated with spironolactone on top of ramipril, excretion of high molecular weight proteins and low molecular weight proteins was reduced compared to placebo and ACEi monotherapy during disease progression (Figure 2B). High molecular weight proteins such as immunoglobulins account for glomerular damage or leakage and low molecular weight proteins such as microglobulins are a sign for tubular damage. In conclusion, dual RAAS-blockade by adding spirolonactone on top of ACEi appeared to have a greater potential in preserving glomerular and tubular function in our mice during the course of AS.

Untreated mice (Plac) showed a remarkable increase in their BUN levels at 65 days, representing the very advanced stage of AS close to end-stage renal failure (Figure 2C). Ramipril monotherapy decreased the BUN levels significantly at 75 and 100 days (compared vs. placebo). Add on therapy with spironolactone on top of ramipril decreased BUN even further, but not significantly different to ACEi-monotherapy, at 75 days and also at 100 days of age (Figure 2C). In the ACEi+Spiro group, two mice died prematurely prior to BUN measurement on day 100, which might reflect an increased risk for hyperkalemia in this group. In conclusion, dual RAAS-blockade appeared to preserve the kidney’s detoxification function better than ACEi monotherapy.

### 3.3. Effect on Matrix Accumulation and Fibrosis during Pathogenesis of Alport Syndrome

**Figure 3:**
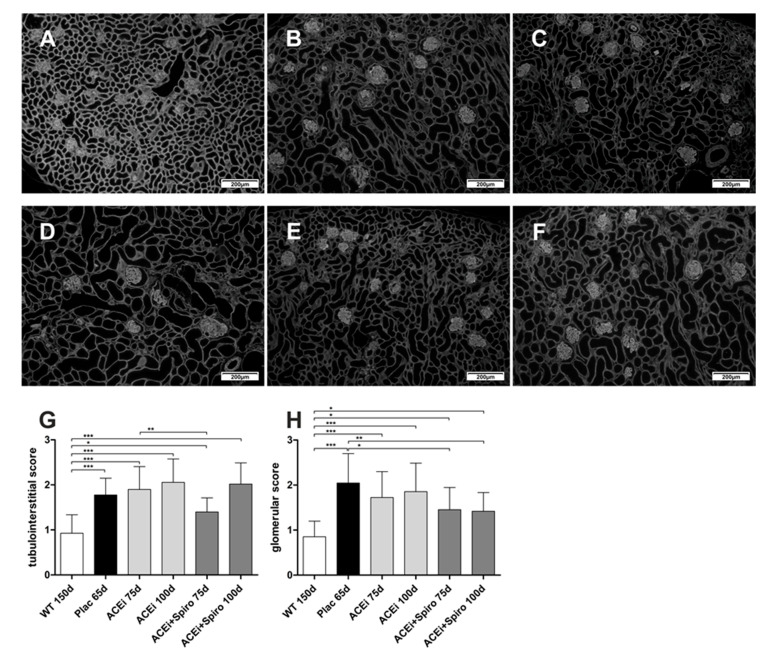
Therapeutic effect on matrix accumulation during pathogenesis of Alport Syndrome (AS). Immune-fluorescence staining of laminin 111, magnification 100-fold. (**A**) wildtype control; (**B**) Alport mice, ACEi 75 days; (**C**) Alport mice, ACEi 100 days; (**D**) Alport mice, Plac (untreated); (**E**) Alport mice, ACEi+Spiro 75 days; (**F**) Alport mice, ACEi-Spiro 100 days; (**G**) score for tubulointerstitial matrix accumulation (*n* = 3 for each group); (**H**) score for glomerular matrix accumulation (*n* = 3 for each group); *** *p* < 0.001; ** *p* < 0.01; * *p* < 0.05.

Tubulointerstitial and glomerular matrix accumulation (laminin 111) was significantly increased in ACEi-treated and untreated Alport mice (Figure 3B–D) compared to wild type mice (Figure 3A; *p* < 0.001). In contrast, when spironolactone was added on top of ramipril, tubulointerstitial matrix accumulation was significantly improved at 75 days compared to ACEi treated mice (*p* < 0.01), an effect which was not maintained until day 100 (Figure 3G). The glomerular extracellular matrix accumulation improved significantly by dual RAAS blockade (ACEi+Spiro; Figure 3E,F) compared to untreated Alport mice at 75 and 100 days of age (*p* < 0.05 and *p* < 0.01) (Figure 3H). In conclusion, dual RAAS-blockade appeared to have a greater inhibitory effect on excessive matrix production in AS than ACEi monotherapy.

**Figure 4:**
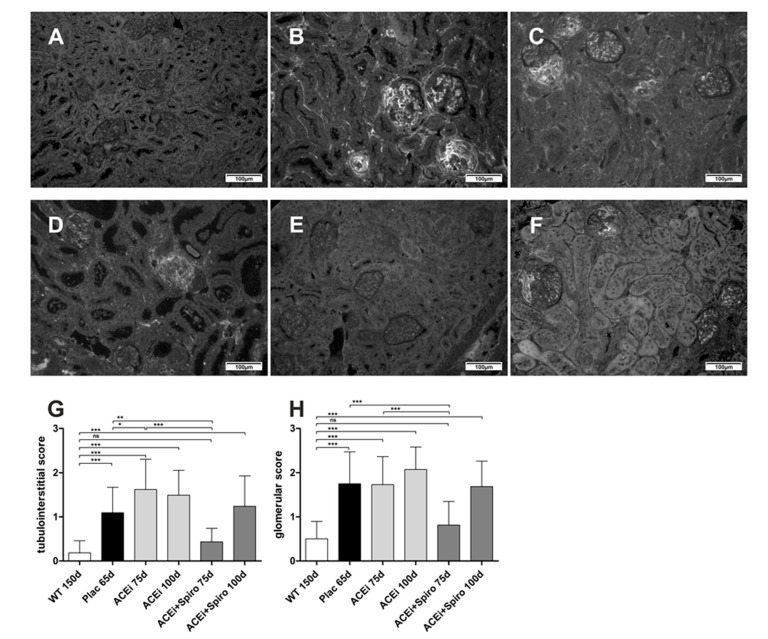
Therapeutic effect on scar tissue formation during pathogenesis of AS. Immune-fluorescence staining of fibronectin, magnification 200-fold. (**A**) wildtype control; (**B**) Alport mice, ACEi 75 days; (**C**) Alport mice, ACEi 100 days; (**D**) Alport mice, Plac (untreated); (**E**) Alport mice, ACEi+Spiro 75 days; (**F**) Alport mice, ACEi-Spiro 100 days; (**G**) score of tubulointerstitial fibrosis (*n* = 3 for each group); (**H**) score of glomerulosclerosis and fibrosis (*n* = 3 for each group); *** *p* < 0.001; ** *p* < 0.01; * *p* < 0.05.

Accelerated scar-tissue formation due to Alport syndrome was determined by fibronectin-staining in each group (Figure 4). Compared to wild type mice, tubulointerstitial and glomerular scarring was severely and significantly increased in untreated Alport mice (Plac) (Figure 4A,D; *p* < 0.001). Adding spironolactone on top of ramipril, significantly improved tubulointerstitial and glomerular at 75 days of age compared to ACEi treated mice (*p* < 0.001) and untreated mice (*p* < 0.01). Similar to matrix accumulation at day 100 (Figure 3G), the significance of this antifibrotic effect at day 75 could not be maintained until day 100 (Figure 4C,F). In conclusion, dual RAAS-blockade appeared to have a greater antifibrotic effect on glomerular and tubulointerstitial scarring in AS than ACEi monotherapy.

### 3.4. Effect on Lifespan until Death Prior to End-Stage Renal Failure in Alport Mice

**Figure 5:**
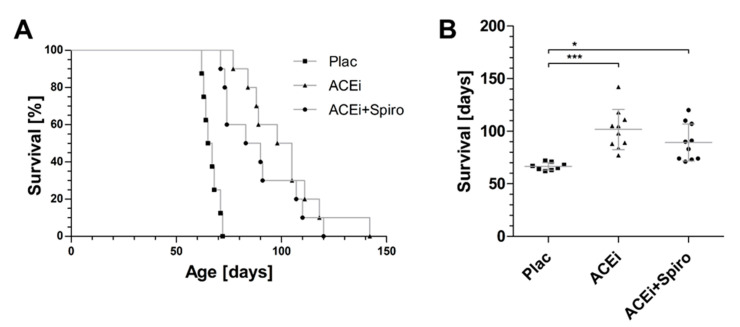
Therapeutic effect on timespan until end-stage renal failure. (**A**) Kaplan–Meier curves of lifespan in days until end-stage renal failure in untreated (Plac), ACEi treated and Alport mice with dual RAAS-blockade; (**B**) Box-blot of lifespan in untreated (Plac), ACEi treated and ACEi+Spiro treated Alport mice. *n* = 8 for Plac, *n* = 10 for ACEi and *n* = 10 for ACEi+Spiro; *** *p* < 0.001; * *p* < 0.05.

In untreated *COL4A3−/−* mice (Plac), mean lifespan until death prior to end-stage renal failure (ESRF) was 66.5 ± 3.7 days (Figure 5). ACEi monotherapy improved lifespan to 101.7 ± 19.1 days. Despite superior BUN levels and improved antiproteinuric and antifibrotic potential of dual blockade (as shown in Figure 2, Figure 3 and Figure 4), lifespan prior to end-stage renal failure was not significantly improved by dual therapy (ACE+Spiro) with a mean survival of 89.3 ± 17.6 days (Figure 5). In conclusion, the improved kidney histology and kidney function parameters of ACEi+Spiro dual therapy were not reflected in better lifespan.

### 3.5. Side-Effects of Dual RAAS-Blockade in Alport Mice

**Figure 6:**
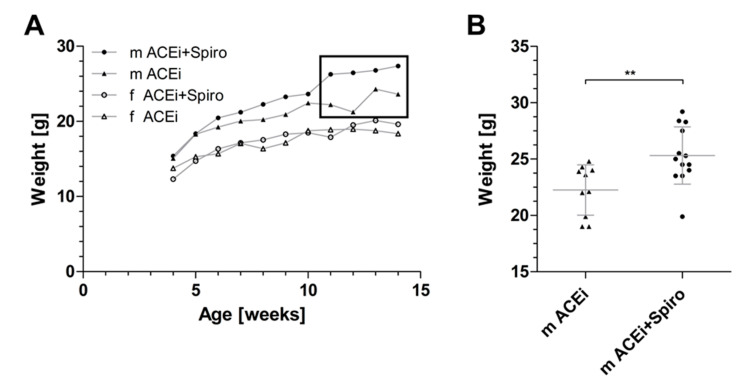
Anti-androgen side-effects of spironolactone on body weight. (**A**) Mean body weight of male and female Alport mice in the ACEi group vs. dual treated mice (ACEi+Spiro); (**B**) Individual measures of body weight of fully grown adult male mice in the ACEi group vs. dual treated males (ACEi+Spiro) as shown in the box in graph A (*n* = 10–13 datapoints for each group). m = male; f = female; ** *p* < 0.01.

The hyperkalemic side-effects of spironolactone under dual RAAS-blockade have been well described in humans, as well as the anti-androgen effects in males [18]. Similarly, the mean weight of male mice with dual therapy (ACEi+Spiro) increased significantly three weeks after initiation of spironolactone treatment (25.3 ± 2.5 g vs. ACEi monotherapy with 22.3 ± 2.2 g; Figure 6).

The body weights of female mice were comparable in both treatment groups. The procedure of taking intra-cardial blood in non-heart-beating sacrificed mice is too susceptible to hyperkalemia in order to generate reliable values. Therefore, our study can only speculate about hyperkalemia as a possible side effect in dual treated Alport mice. In conclusion, dual RAAS-blockade by adding spironolactone on top of ACEi appeared to be associated with common and well-known side effects such as increasing body weight in male mice due to anti-androgen effect of spironolactone and, possibly, hyperkalemia.

## 4. Discussion

ACEis delay renal failure in patients with AS by many years [12]. However, over the long course of treatment, some of the antiproteinuric (and nephroprotective) effects of ACEis seem to weaken in a relevant number of patients. In the present study, increased levels of plasma aldosterone after long-term ACEi therapy, known as “aldosterone escape” [16,17], were hypothesized to be an important negative effect on disease progression in AS. Therefore, the present study investigates if the aldosterone antagonist spironolactone can counteract this loss of ACEi-efficacy in the Alport mouse model.

According to the low aldosterone levels (close to wildtype-controls) in our ACEi+Spiro group, dual therapy could be effective: dual therapy revealed in lower BUN levels, less proteinuria, less matrix accumulation, and less fibrosis than ACEi monotherapy. However, lifespan until end-stage renal failure was not improved. Recent data showed that the use of spironolactone in CKD patients is associated with a lower risk of ESRD, but also a higher risk of hyperkalemia-related hospitalization, indicating that hyperkalemia should be closely monitored during spironolactone therapy [18]. The incidence rate of severe hyperkalemia was about 6.5% in patients with an eGFR < 60 mL/min/1.73 m^2^, but zero in patients with eGFR ≥ 60 mL/min/1.73 m^2^ during dual RAAS-blockade with ACEi or ARB plus spironolactone. These data suggest that spironolactone-associated severe side effects (such as hyperkalemia) are more likely to occur in patients with CKD stages 3 and 4 [19]. In addition, dual RAAS-blockade with spironolactone resulted in a significant drop in blood pressure (−7 mmHg diastolic, −12 mmHg systolic blood pressure), loss of eGFR (−9.3 mL/min/1.73 m^2^) and a significant decrease of albuminuria (−57%) in diabetic patients after 24 weeks of treatment with spironolactone on top of ACEi or ARB [20]. Therefore, in our study, the beneficial antiproteinuric, antifibrotic effects of dual RAAS-blockade with spironolactone might not have resulted in improved lifespan prior to ESRF because of premature death due to severe side effects of dual RAAS-blockade such as hyperkalemia.

Hematuria is the earliest manifestation of Alport syndrome in the kidney, followed by microalbuminuria proceeding to overt proteinuria, impaired kidney function, finally leading to ESRF. Therefore, proteinuria and albuminuria are very important indicators of the severity of Alport syndrome and disease progression. In our study, adding spironolactone on top of ACEi decreased proteinuria and kept the level of proteinuria rather stable until late stages (at 100 days of age) compared to ACEi monotherapy. Further analysis of urine proteins showed that dual therapy did better in reducing high molecular weight proteins (as a sign for glomerular damage) and low molecular weight proteins (as a sign for tubular damage), suggesting that spironolactone had a protective effect on glomerular and tubular function. In parallel, tubular-interstitial and glomerular matrix accumulation were significantly reduced in ACEi+Spiro treated mice compared to ACEi monotherapy. As limitation of our study, the diuretic effect of spironolactone in the ACEi+Spiro group may have increased urine volume and reduced urinary protein concentration of spot urine. Glomerular and tubulointerstitial scar tissue formation was significantly reduced in dual treated mice compared to placebo and ACEi monotherapy. Therefore, our study showed a meaningful improvement in preserving kidney structure and function in the ACEi+Spiro group compared to ACEi-monotherapy in mice with Alport syndrome. Similar results of renoprotective effects from spironolactone add-on therapy have previously been reported in patients with albuminuria, including diabetic nephropathy [21,22]. In addition, patients with heart failure and renal dysfunction, spironolactone therapy has been shown to improve the two-year survival despite worsening eGFR and elevated potassium levels [23]. A small proof-of-concept study in five patients with AS, treated with spironolactone added after long term use of ACEi and ARBs, showed a significant reduction of the urinary protein/creatinine ratio at 3, 6, 12 and 18 months [24].

Elevated levels of plasma aldosterone significantly correlate with the decline of the glomerular filtration rate and higher risk of ESRF [25,26]. Our experiments were powered for the “primary end-point” end-stage renal failure, not for serum levels of aldosterone. This limits the informative value of the measurements of the aldosterone level, which were actually only intended as a further proof of concept. In our study with a very limited number of mice and aldosterone blood tests, ACEi+Spiro dual therapy resulted in constant low levels of serum aldosterone close to wildtype controls without CKD. Serum aldosterone levels were elevated in untreated Alport mice as well as in older Alport mice with long-lasting ACEi monotherapy, mimicking an “aldosterone-escape”. Therefore, lower aldosterone levels in older Alport mice on dual therapy are the likely cause for improved kidney structure and function. However, Kim and coworkers reported that proteinuria reduction was not associated with the serum aldosterone concentration and add-on spironolactone treatment was more effective in the aldosterone non-escape group compared to the aldosterone escape group of CKD patients [19]. Recently, spironolactone was reported to be able to ameliorate endothelial dysfunction through intracellular oxidative stress attenuation in a 5/6 nephrectomy rat model [27]. It has been reported that spironolactone rescued renal dysfunction in obstructive jaundice rats by upregulating angiotensin-converting enzyme 2 expression, an important protective factor in diabetic nephropathy [28]. These data point to additional nephroprotective mechanisms of spironolactone despite its capability to reduce aldosterone levels, which should be explored in further studies [29,30].

## 5. Conclusions

In our mouse model of Alport syndrome, spironolactone on top of ACEi is superior in preserving kidney function, kidney architecture, and delaying renal fibrosis. However, in our mouse model, the nephroprotective effect of dual RAAS-blockade was counteracted by premature death possibly due to a higher risk of serious side effects, therefore not improving the lifespan of Alport mice until ESRF. Therefore, dual RAAS-blockade by adding spironolactone could offer an effective therapy option for patients with Alport syndrome, who experience a loss of the antiproteinuric effect after long ACEi therapy due to an aldosterone-escape, which can be diagnosed by high aldosterone levels. Yet, the increased risk of potential side effects should be very seriously taken into account before starting dual RAAS-blockade. Dual RAAS-blockade requires close monitoring and strict safety measures, possibly including potassium-sparing diet or even potassium-binders added to the standard medication.

## Data Availability

Data supporting reported results can be requested from the corresponding author.
